# Supplementary material for: Multiplexed Digital Spatial Protein Profiling Reveals Distinct Phenotypes of Mononuclear Phagocytes in Livers with Advanced Fibrosis
Source: Cells. 2022 Oct 26;11(21):3387. doi: 10.3390/cells11213387 (PMC9654480; doi:10.3390/cells11213387)

Supplementary Table S1. Multivariate logistic regression analysis of other protein signatures associated with high-grade fibrosis.

	Multivariable logistic regression			
	Variable	**coefficient**	**Odds ratio (95% CI)**	**p-value**
**Model 1**	ARG1_B7.H3_CD127_CD68_HLA.DR_OX40L_pan.RAS_STING_Tim.3	4.525	92.26 (10.50–810.67)	4.49 x10^{-5}
	Age (≤55 years vs >55 years)	-0.783	0.46 (0.09–2.32)	0.345
	BMI (≤25 kg/m^2 vs >25 kg/m^2)	2.602	13.49 (1.40–130.23)	0.025
**Model 2**	B7.H3_CD68_HLA.DR_OX40L_Phospho.c.RAF_STING_VISTA	4.525	92.26 (10.50–810.67)	4.49 x10^{-5}
	Age (≤55 years vs >55 years)	-0.783	0.46 (0.09–2.32)	0.345
	BMI (≤25 kg/m^2 vs >25 kg/m^2)	2.602	13.49 (1.40–130.23)	0.025
**Model 3**	Beta.2.microglobulin_CD127_CD68_HLA.DR_OX40L_Tim.3	4.762	116.94 (12.27–1114.37)	3.48 x10^{-5}
	Age (≤55 years vs >55 years)	-1.108	0.33 (0.06–1.80)	0.200
	BMI (≤25 kg/m^2 vs >25 kg/m^2)	2.786	16.22 (1.65–159.51)	0.017
**Model 4**	CD68_HLA.DR_OX40L_pan.RAS_STING	4.6492	104.50 (11.40–957.83)	3.91 x10^{-5}
	Age (≤55 years vs >55 years)	-0.5631	0.57 (0.11–3.06)	0.5115
	BMI (≤25 kg/m^2 vs >25 kg/m^2)	2.7700	15.96 (1.64–155.03)	0.0170

Supplementary Figure S1. Performance evaluation of the protein signature in the (A) CHB subgruop (n=46) and (B) non-CHB group (n=18)

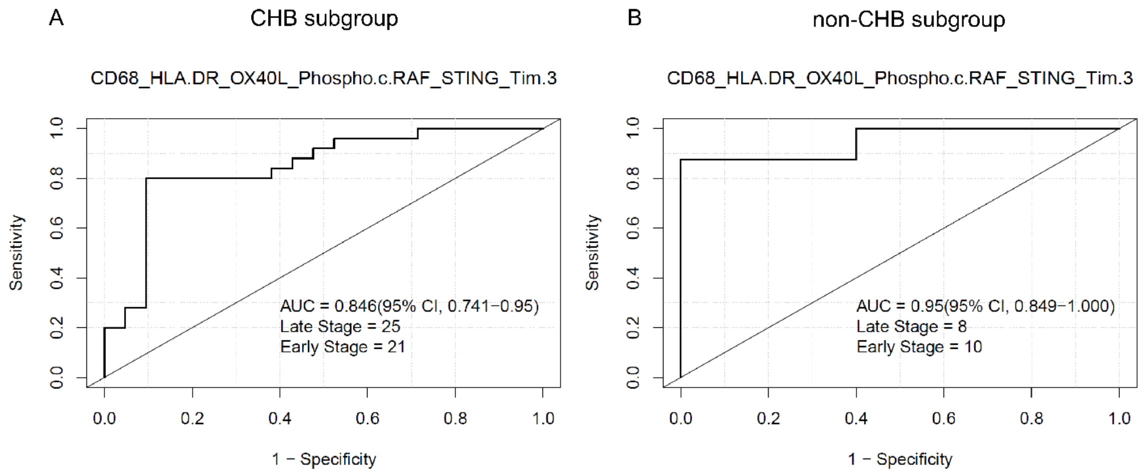

Supplement: Supplementary file 1 [file cells-11-03387-s001.zip › cells-1930563-supplementary.pdf]
